# Clinical REsearch During Outbreaks (CREDO) Training for Low- and Middle-Income Countries

**DOI:** 10.3201/eid2511.180628

**Published:** 2019-11

**Authors:** Nzelle Delphine Kayem, Amanda Rojek, Emmanuelle Denis, Alex Salam, Andreas Reis, Piero Olliaro, Peter Horby

**Affiliations:** University of Oxford, Oxford, UK (N.D. Kayem, A. Rojek, E. Denis, A. Salam, P. Olliaro, P. Horby);; United Kingdom Public Health Rapid Support Team, Oxford (A. Salam);; World Health Organization, Geneva, Switzerland (A. Reis)

**Keywords:** capacity building, research design, epidemics, curriculum, low- and middle-income countries, LMICs, outbreaks, Clinical REsearch During Outbreaks, CREDO

## Abstract

We describe a pilot of the Clinical REsearch During Outbreaks (CREDO) initiative, a training curriculum for researchers in epidemic-prone low- and middle-income countries who may respond to disease outbreaks. Participants reported improved confidence in their ability to conduct such research and overall satisfaction with the course structure, content, and training.

Clinical research is an essential component of outbreak response because it underpins clinical management and public health measures (*1*). However, conducting clinical research during epidemics is challenging (*2*,*3*). The Ebola epidemic in West Africa underscored the need for improved research infrastructure and capacity in epidemic-prone vulnerable low- and middle-income countries (LMICs) (*4*). To address this need, a training curriculum, Clinical REsearch During Outbreaks (CREDO), was jointly developed by the Special Programme for Research and Training in Tropical Diseases (TDR), hosted by the World Health Organization, the International Severe Acute Respiratory and Emerging Infections Consortium, and the United Kingdom Public Health Rapid Support Team. The main objective of the CREDO training curriculum is to strengthen the national capacities of LMICs to design and implement clinical research during outbreaks of infectious diseases and to improve team capacity and team effort. We describe the development and piloting of the CREDO training curriculum and present the results of the formal evaluation.

## The Study

The learning objectives for the CREDO curriculum (Table 1) were developed using the TDR Global Competency Framework for Clinical Research (*5*,*6*), which describes all areas of competency required to conduct clinical research (*6*). The content of the modules addressed both observational studies and clinical trials. Observational studies were included because they are essential for understanding the etiology, natural history, and pathophysiology of epidemic infectious diseases, many of which are poorly understood. Clinical trials address questions on therapeutics, diagnostics, and other topics, and CREDO covers a range of trial designs including adaptive designs, which may be better suited to the evolving nature of outbreaks.

**Table 1:** Learning objectives for the Clinical REsearch During Outbreaks (CREDO) training course

No.	Objective
1.	Define what emerging and epidemic infections are and discuss their importance
2.	Critique the clinical research response to emerging infections
3.	Understand the key elements in a rapid systematic review
4.	Critically appraise literature and identify gaps in the literature
5.	Select an appropriate study design
6.	Outline the ethical implications of the selection of study design
7.	Describe the ethical considerations required to ensure that informed consent is obtained, particularly in traditional communities or low-resource settings
8.	Describe ethical principles of incorporating special groups (pregnant women, children, etc.) in research during epidemics
9.	Describe ways of minimizing participant loss to follow-up
10.	Identify logistical and operational factors affecting the implementation of clinical research during an outbreak
11.	Formulate project management timelines for a research project
12.	Plan efficient data collection methods
13.	Describe special considerations for community engagement in outbreak research
14.	Assemble a communications team and develop a crisis communications plan
15.	Identify potential sources of study funding and prepare grant applications
16.	Identify a study sponsor and describe the role and responsibilities of the sponsor
17.	Describe the basic elements of the different kinds of contracts used in clinical research
18.	Explain the benefits of streamlined data collection
19.	Explain the important role of data sharing, harmonization, and collaboration in outbreak research

CREDO is structured as a blended-learning format, with face-to-face sessions (workshops) and asynchronous, downloadable online sessions (e-learning). Our training model was designed with the workshops occurring before and after the e-learning component to introduce and then solidify knowledge gained in the online component. Our assessment model combined multiple-choice questions in the e-learning sessions with simulation exercises in the workshop sessions to provide a balance in testing between recall and critical or creative thinking (*7*).

The curriculum has 12 modules (Table 2): 1 workshop and 11 e-learning modules, 2 of which are prerequisites that must be completed before the workshop date. Modules are freely available online through a digital platform suited to the low bandwidths found in LMICs. The e-learning component is hosted on the Global Health Network website (https://isaric.tghn.org/credo). It is self-paced over a period of 5–6 months, and each module takes an average of 1–2 hours, with some taking as many as 4–5 hours, to complete. The e-learning is completed individually; participants complete the final assessment during the second workshop as a team, each team member actively using the knowledge learned from the online modules to contribute to the design of a clinical trial during a hypothetical outbreak. Each participant who successfully completes all course components earns a CREDO certificate. 

**Table 2:** Curriculum structure of Clinical REsearch During Outbreaks (CREDO) training course

Module title	Module summary and website link
Prerequisite modules	
Good clinical practice	A framework of principles to ensure the safety of research participants and integrity and validity of data: https://globalhealthtrainingcentre.tghn.org/ich-good-clinical-practice
The global health research process map	A pragmatic interactive tool provides step-by-step guidance for each stage that needs to be considered when planning a new study: https://processmap.tghn.org/about
Workshop: Evidence-based medicine for epidemic infections and key issues in study design	Delivered in 3 presentations, it provides an introduction to epidemic and emerging infections, critiques the clinical research responses to previous outbreaks, highlights the challenges to clinical research during outbreaks, and discusses possible mitigating strategies: https://isaric.tghn.org/credo/credo-workshop-1
E-learning component: self-paced, in any order, completed individually before the second workshop	
Rapid evidence-needs appraisal	A guide for the conduct of rapid reviews in the event of an outbreak: https://globalhealthtrainingcentre.tghn.org/credo-rapid-evidence-needs-appraisal
Research study planning and governance	An overview of how to set up a clinical research study and find and apply for funding, and important concepts for study managements: https://globalhealthtrainingcentre.tghn.org/credo-research-study-planning-and-governance
Study design	An introduction to the challenges to research design during outbreaks and some mitigating strategies: https://globalhealthtrainingcentre.tghn.org/credo-study-design
Statistics	Background of statistical principles relevant to clinical research and trial design and challenges in outbreaks and some solutions: https://globalhealthtrainingcentre.tghn.org/credo-statistics
Logistics and operational planning	Pragmatic solutions to common logistical and operational challenges to research in outbreaks: https://globalhealthtrainingcentre.tghn.org/credo-logistical-and-operational-planning
Data sharing and harmonization	General outline of data management, sharing, and harmonization to guide the conduct of research during outbreaks: https://isaric.tghn.org/credo/credo-data-sharing-and-harmonisation
Ethics	WHO course on ethics in outbreaks; modules 2, 4, and 6 on the TGHN platform: https://globalhealthtrainingcentre.tghn.org/research-ethics-epidemics-pandemics-and-disaster-situations
Communications and engagement	Effective communication and engagement during outbreaks: https://globalhealthtrainingcentre.tghn.org/credo-communications-and-community-engagement
Special groups: Children, pregnant women, mother/child	A consensus statement on the inclusion of children and pregnant women in research in disease outbreaks to help facilitate including these important groups in future research and clinical trials: https://isaric.tghn.org/credo/ethics-special-groups
Workshop: final assessment, completed in teams	

*Prerequisite modules must be completed online individually before the first workshop. TGHN, The Global Health Network; WHO, World Health Organization.

We designed the curriculum for team training with multidisciplinary and intact teams (*8*); that is, teams that are already working together to develop and implement clinical studies. Team training refers to the training of an entire team and has resulted in improved patient outcomes in various areas of medicine, such as trauma (*8*,*9*).

During March–August 2017, we conducted a pilot of the CREDO training curriculum. An informal call invited team applications from sub-Saharan Africa, targeted because populations in the region are at risk for outbreaks from high-threat infectious diseases (*10*). We selected 19 participants from 4 multidisciplinary teams in 4 countries (Ethiopia, Ghana, Côte d’Ivoire, and Uganda) on the basis of their clinical research experience and team diversity. Of the selected participants, 8 were female and 11 male. Teams consisted of 11 medical doctors, 2 research nurses, 2 data managers, 1 clinical trial manager, and 3 biomedical scientists.

We conducted the first workshop in Entebbe, Uganda. Its purpose was to appraise the challenges of outbreak research; describe the fundamental concepts of generating clinical evidence; introduce the online component of the training; and assess, using simulated team-based exercises, the participants’ confidence in planning and conducting clinical research during an outbreak. After the workshop, participants had time to complete the e-learning modules.

The closing workshop was conducted in Addis Ababa, Ethiopia. Two weeks before this workshop, we gave participants an assignment to develop a clinical trial protocol for a hypothetical outbreak (available on the course website, https://isaric.tghn.org/credo/credo-overall-assignment). This workshop increased and assessed the participants’ knowledge and understanding of observational research and clinical trials. The clinical trial protocols in the assignment were assessed using role play; facilitators played the roles of statisticians, community representatives, scientific experts, ethical reviewers, and government representatives. The teams then incorporated suggestions made by the faculty. The revised protocols then underwent review by peers using an adapted scoring tool (Appendix).

Pilot participants completed an online pre-course and post-course evaluation form anonymously. The questions were based on the learning objectives (Table 1) and sought the participants’ views on the training received, the structure and the content of the individual course modules, and their ability to conduct clinical research during an outbreak. The responses were structured on a Likert scale from strongly agree to strongly disagree or not confident to very confident, with free-text space to clarify any responses (Appendix).

**Figure 1:**
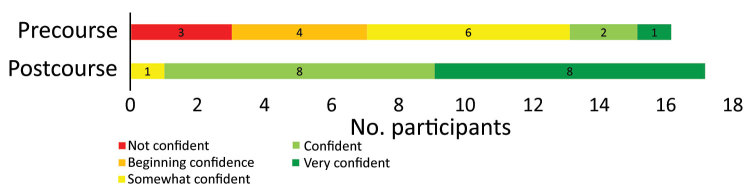
Self-assessed level of confidence with learning objectives of Clinical REsearch During Outbreaks (CREDO) before and after course. Participants’ level of confidence in their ability to implement a clinical research study during an outbreak changed substantially: in the precourse assessment, 3 of 17 participants rated themselves as confident or very confident; in postcourse assessment, 16 of 17 did.

Of the 19 participants, 16 (84.2%) completed the precourse questionnaire and 17 (89.5%) completed the postcourse questionnaire. Most participants were satisfied with the course; 16 participants rated their level of satisfaction as satisfied or very satisfied. Self-assessed levels of confidence increased after the course; 16 participants rated their level of confidence in implementing the course objectives as confident or very confident after the course, compared with only 3 participants before the course (Figure 1). However, another evaluation is required during or after an outbreak to better assess the effectiveness of the course.

**Figure 2:**
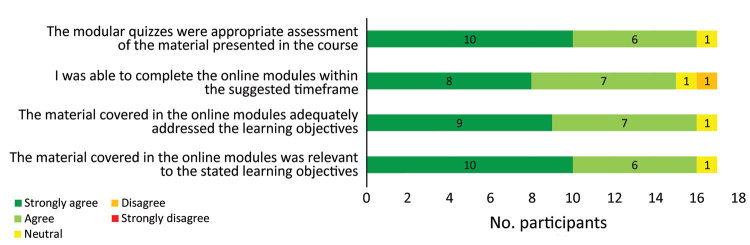
Participant level of agreement with postcourse assessment questions about the quality of the online component of the Clinical REsearch During Outbreaks (CREDO) curriculum.

An assessment of the content of CREDO showed largely positive reviews, particularly with respect to the relevance and adequacy of the material covered in each of the course modules (Figure 2). Time for completion of each module was generally sufficient except in the rapid evidence appraisal module, for which 3 participants indicated time was inadequate. Some of the major strengths of CREDO were its structure and content, which most participants considered “holistic” and “comprehensive.”

## Conclusions

Although clinical research is becoming recognized as an important component of outbreak management (*1*,*11*), few resources exist to assess or build capacity in this area. Most of the available outbreak training programs or courses focus on surveillance, epidemiology, outbreak investigation, or laboratory investigation (*12*–*15*). CREDO offers a unique perspective with its focus on clinical research and clinical trials.

The strengths of the CREDO training curriculum lie in its format of blended learning, team-based approach, and content tailored to outbreak conditions. Its combination of training in observational studies and clinical trials builds capacity for collecting the much-needed information on pathophysiology and natural history of poorly known conditions, essential components to identify the right interventions to test and the design of the intervention trials.

In addition, CREDO, as an open-access resource, lends itself to adoption and adaptation by interested sites and to creating a community of science by sharing additional or modified materials through the Global Health Network website. CREDO resources can be incorporated into medical or public health training curricula or used as a standalone course for continuous professional development. Some ways in which CREDO will become sustainable are by broadening the number of trainers from among course participants, supporting country ownership, and franchising the course through a variety of providers.

Appendix Example of peer review form and questionnaire for pre- and post-course evaluation of Clinical REsearch During Outbreaks (CREDO) course.
