# Supplementary material for: Clinical REsearch During Outbreaks (CREDO) Training for Low- and Middle-Income Countries
Source: Emerg Infect Dis. 2019 Nov;25(11):2084–7. doi: 10.3201/eid2511.180628 (PMC6810185; doi:10.3201/eid2511.180628)
Supplement: Appendix — Example of peer review form and questionnaire for pre- and post-course evaluation of Clinical REsearch During Outbreaks (CREDO) course. [file 18-0628-Techapp-s1.pdf]

Article DOI: https://doi.org/10.3201/eid2511.180628

Clinical REsearch During Outbreaks (CREDO) Training for Low- and Middle-Income Countries

Appendix

The following pages show the scoring tool used to assess participants’ work during the CREDO training program and the evaluation form given to participants after completion of the training program.

CREDO
CLINICAL REsEARCH DURING OUTBREAKS

GROUP UNDER REVIEW: ____________

For each criterion please give a score of 1 to 5. 1 = poor or unsatisfactory 5=excellent

	Criteria	Explanation	Score	Why you gave this score
1.	**Importance/Potential**	The primary outcome measure is clinically relevant		
		The primary outcome measure is clearly defined		
2.	**Feasibility**	Operational issues are identified and adequately addressed		
		The project is likely to be delivered within the time frame suggested		
3.	**Ethics**	Ethical issues are identified and adequately addressed		
		Measures are in place to ensure patient safety and welfare		
4.	**Study design**	The trial design is appropriate for the question		
		The design is robust to potential uncertainties of the outbreak		
5.	**Statistical soundness** – **Has the applicant clearly set out and justified the following:**	Measures for avoidance of bias (e.g. blinding, randomisation)		
		Number of experimental and control groups and sample size per group		
		Overview of the planned statistical analyses in relation to the primary outcomes to be assessed		
		Frequency of measurements/interventions to be used		

CREDO Course Evaluation

*Required

CLINICAL RESEARCH DURING OUTBREAKS

Post - Course Evaluation

In order to gauge the success of the CREDO pilot we require all participants to fill in a post-course evaluation form. Your answers will be anonymised so please try to answer as accurately and honestly as possible.

1. Main job role: *

   Mark only one oval.

   - Principal Investigator
   - Medical Doctor
   - Research Nurse
   - Clinical Trial Manager
   - Biomedical scientist
   - Clinical Data Manager
   - Pharmacist
   - Other: ____________________

2. General Research Experience (years) *

   ____

3. Clinical Research Experience (years) *

   ____

4. Experience with Outbreak Research (years) *

   ____

CREDO learning objectives

5. The CREDO learning objectives are listed below. Now that you have completed the training, please indicate your level of confidence for each of the following. *

Mark only one oval per row.

	Not Confident	Beginning Confidence	Somewhat Confident	Confident	Very Confident
Define what emerging and epidemic infections are and discuss their importance	⬭	⬭	⬭	⬭	⬭
Critique the clinical research response to emerging infections.	⬭	⬭	⬭	⬭	⬭
Understand the key elements in a rapid systematic review	⬭	⬭	⬭	⬭	⬭
Critically appraise literature and identify gaps in literature	⬭	⬭	⬭	⬭	⬭
Select an appropriate study design	⬭	⬭	⬭	⬭	⬭
Outline the ethical implications of the selection of study design	⬭	⬭	⬭	⬭	⬭
Describe the ethical considerations required to ensure that informed consent is obtained, particularly in traditional communities or low resource settings	⬭	⬭	⬭	⬭	⬭
Describe ethical principles of incorporating special groups (pregnant women, children etc.) in research during epidemics	⬭	⬭	⬭	⬭	⬭
Describe ways of minimising participant loss to follow up	⬭	⬭	⬭	⬭	⬭
Identify logistical and operational factors affecting implementation of clinical research during an outbreak	⬭	⬭	⬭	⬭	⬭
Formulate project management timelines for a research project	⬭	⬭	⬭	⬭	⬭
Plan efficient data collection methods	⬭	⬭	⬭	⬭	⬭
Describe special considerations for community engagement in outbreak research	⬭	⬭	⬭	⬭	⬭
Assemble a communications team and develop a crisis communications plan	⬭	⬭	⬭	⬭	⬭
Identify potential sources of study funding and prepare grant applications	⬭	⬭	⬭	⬭	⬭

	Not Confident	Beginning Confidence	Somewhat Confident	Confident	Very Confident
Identify a study Sponsor and describe the role and responsibilities of the Sponsor					
Describe the basic elements of the different kinds of contracts used in clinical research					
Explain the benefits of streamlined data collection					
Explain the important role of data sharing, harmonisation and collaboration in outbreak research					

Evaluation of CREDO online modules

6. For the Rapid 'Evidence Needs' appraisal module, please indicate your level of agreement with the following statements: *

Mark only one oval per row.

	Strongly disagree	Disagree	Neutral	Agree	Strongly agree
The material covered in the module was relevant to the stated learning objectives.					
The material covered in the module adequately addressed the learning objectives.					
I was able to complete the module within the suggested time-frame					
The final quiz was an appropriate assessment of the material presented in the course.					

7. If you answered 'disagree' or 'strongly disagree' to any of the above please let us know why.

____

____

____

____

____

8. For the Data Sharing and Harmonisation module, please indicate your level of agreement with the following statements: *

Mark only one oval per row.

	Strongly disagree	Disagree	Neutral	Agree	Strongly agree
The material covered in the module was relevant to the stated learning objectives.	◯	◯	◯	◯	◯
The material covered in the module adequately addressed the learning objectives.	◯	◯	◯	◯	◯
I was able to complete the module within the suggested time-frame	◯	◯	◯	◯	◯
The final quiz was an appropriate assessment of the material presented in the course.	◯	◯	◯	◯	◯

9. If you answered 'disagree' or 'strongly disagree' to any of the above please let us know why.

____

____

____

____

____

10. For the Logistical and Operational Planning module, please indicate your level of agreement with the following statements: *

Mark only one oval per row.

	Strongly disagree	Disagree	Neutral	Agree	Strongly agree
The material covered in the module was relevant to the stated learning objectives.	◯	◯	◯	◯	◯
The material covered in the module adequately addressed the learning objectives.	◯	◯	◯	◯	◯
I was able to complete the module within the suggested time-frame	◯	◯	◯	◯	◯
The final quiz was an appropriate assessment of the material presented in the course.	◯	◯	◯	◯	◯

11. If you answered 'disagree' or 'strongly disagree' to any of the above please let us know why.

____

____

____

____

____

12. For the Research Study Planning and Governance module, please indicate your level of agreement with the following statements: *

Mark only one oval per row.

	Strongly disagree	Disagree	Neutral	Agree	Strongly agree
The material covered in the module was relevant to the stated learning objectives.					
The material covered in the module adequately addressed the learning objectives.					
I was able to complete the module within the suggested time-frame					
The final quiz was an appropriate assessment of the material presented in the course.					

13. If you answered 'disagree' or 'strongly disagree' to any of the above please let us know why.

____

____

____

____

____

14. For the Communications and Community Engagement module, please indicate your level of agreement with the following statements: *

Mark only one oval per row.

	Strongly disagree	Disagree	Neutral	Agree	Strongly agree
The material covered in the module was relevant to the stated learning objectives.					
The material covered in the module adequately addressed the learning objectives.					
I was able to complete the module within the suggested time-frame					
The final quiz was an appropriate assessment of the material presented in the course.					

15. If you answered 'disagree' or 'strongly disagree' to any of the above please let us know why.

____

____

____

____

____

16. For the Ethics in epidemics, emergencies and disasters module, please indicate your level of agreement with the following statements: *

Mark only one oval per row.

	Strongly disagree	Disagree	Neutral	Agree	Strongly agree
The material covered in the module was relevant to the stated learning objectives.					
The material covered in the module adequately addressed the learning objectives.					
I was able to complete the module within the suggested time-frame					
The final quiz was an appropriate assessment of the material presented in the course.					

17. If you answered 'disagree' or 'strongly disagree' to any of the above please let us know why.

____
____
____
____
____

18. For the Study Design module, please indicate your level of agreement with the following statements: *

Mark only one oval per row.

	Strongly disagree	Disagree	Neutral	Agree	Strongly agree
The material covered in the module was relevant to the stated learning objectives.					
The material covered in the module adequately addressed the learning objectives.					
I was able to complete the module within the suggested time-frame					
The final quiz was an appropriate assessment of the material presented in the course.					

19. If you answered 'disagree' or 'strongly disagree' to any of the above please let us know why.

____
____
____
____
____

20. For the Statistics module, please indicate your level of agreement with the following statements: *

Mark only one oval per row.

	Strongly disagree	Disagree	Neutral	Agree	Strongly agree
The material covered in the module was relevant to the stated learning objectives.	⬭	⬭	⬭	⬭	⬭
The material covered in the module adequately addressed the learning objectives.	⬭	⬭	⬭	⬭	⬭
I was able to complete the module within the suggested time-frame	⬭	⬭	⬭	⬭	⬭
The final quiz was an appropriate assessment of the material presented in the course.	⬭	⬭	⬭	⬭	⬭

21. If you answered 'disagree' or 'strongly disagree' to any of the above please let us know why.

____

____

____

____

____

CREDO overall evaluation

22. How satisfied were you with the CREDO training overall? *

Mark only one oval.

	1	2	3	4	5	
Very dissatisfied	⬭	⬭	⬭	⬭	⬭	Very satisfied

23. Please identify what you consider to be the strengths of the CREDO training

____

____

____

____

____

24. What areas of CREDO do you think need improvement?

Thank you for completing this evaluation!

Powered by
Google Forms
